# Association of *Staphylococcus* nasal colonization and HIV in end-stage renal failure patients undergoing peritoneal dialysis

**DOI:** 10.1080/0886022X.2019.1598433

**Published:** 2019-04-17

**Authors:** Kwazi C. Z. Ndlovu, Khine Swe Swe-Han, Alain Assounga

**Affiliations:** a Department of Internal Medicine, Division of Nephrology, University of the Free State, Bloemfontein, South Africa;; b Inkosi Albert Luthuli Central Hospital, Durban, South Africa;; c Department of Medical Microbiology, School of Laboratory Medicine & Medical Science, University of KwaZulu-Natal, Durban, South Africa;; d Department of Nephrology, University of KwaZulu-Natal, Durban, South Africa

**Keywords:** Continuous ambulatory peritoneal dialysis (CAPD), HIV, peritonitis, *Staphylococcus aureus*, nasal carriage, coagulase-negative *Staphylococcus*

## Abstract

**Introduction:** Staphylococcal infections can cause significant morbidity in patients undergoing dialysis. This study evaluated the effects of HIV infection on nasal carriage of *Staphylococcus aureus*, staphylococcal peritonitis, and catheter infection rates in patients with end-stage renal failure managed with continuous ambulatory peritoneal dialysis (CAPD). **Methods:** Sixty HIV-positive and 59 HIV-negative CAPD patients were enrolled and followed up for up to 18 months. *S. aureus* nasal carriage (detected by nasal swab culture), Staphylococcal peritonitis (diagnosed by clinical presentation, and CAPD effluent Staphylococcal culture and white blood cell count ≥100 cells/µL), and catheter infections (including exit site and tunnel infections) were assessed monthly. **Results:** At 18 months, *S. aureus* nasal carriage rates were 43.3% and 30.5% (*p =* 0.147) and the methicillin-resistant *S. aureus* (MRSA) nasal carriage rates were 31.7% and 13.6% (*p =* 0.018) for the HIV-positive and HIV-negative cohorts, respectively. The HIV-positive cohort was associated with increased hazards for staphylococcal peritonitis, (adjusted hazard ratio [AHR] 2.85, 95% confidence interval [CI] 1.19–6.84, *p =* 0.019) due to increased coagulase-negative staphylococcal (CNS) peritonitis rate in the HIV-positive cohort compared with the HIV-negative cohort (0.435 vs. 0.089 episodes/person-years; AHR 7.64, CI 2.18–26.82, *p =* 0.001). On multivariable analysis, CD4+ cell count <200 cells/µL, diabetes, and *S. aureus* nasal carriage were found to be independent predictors of *S. aureus* peritonitis. **Conclusions:** These findings suggest that HIV infection may be a risk factor for MRSA nasal colonization and may increase the risks of CNS peritonitis, while a CD4+ cell count <200 cells/µL and *S. aureus* nasal carriage may be important predictors of *S. aureus* peritonitis.

## Introduction

Infection is a major challenge in patients with end-stage renal failure who are managed with continuous ambulatory peritoneal dialysis (CAPD), and it is an important source of morbidity and technique failure. Gram-positive bacteria, most notably coagulase-negative staphylococci (CNS) and *Staphylococcus aureus*, frequently cause CAPD-associated peritonitis [1–3]. Peritonitis caused by CNS infection tends to follow a benign clinical course that is easily treatable, while *S. aureus* peritonitis can be complicated with relapses and the need for catheter removal, particularly if it is associated with exit site or tunnel infections. An important risk factor for CAPD-associated exit site infections and peritonitis is *S. aureus* nasal carriage [4–6]. An association between HIV infection and increased rates of *S. aureus* nasal colonization in the general population has been reported, and increased likelihood of colonization has been suggested in the advanced stages of HIV infection [7,8].

Infective complications in HIV-positive patients on dialysis can cause significant morbidity and mortality, and they can result in the need to transfer to hemodialysis, which gives rise to greater cost burdens to health budgets. In poorly resourced regions, such as sub-Saharan Africa, where HIV is prevalent, but access to renal replacement therapy (RRT) is limited, CAPD can represent a cost-effective option. Africa has been estimated to have the lowest RRT access at between 9% and 16%, reflecting a significant unmet need [9]. Indeed, CAPD can be implemented with relative ease and without the need for complex equipment, and it is well suited for areas that are remote or have limited dialysis facilities [10–12]. This study aimed to evaluate the effects of HIV infection on *S. aureus* nasal carriage, staphylococcal peritonitis, and catheter infection rates in patients with end-stage renal disease (ESRD) who were managed with CAPD.

## Materials and methods

### Study population

This prospective sub-cohort of 129 patients was drawn from a 140-patient cohort recruited from King Edward VIII Hospital and Inkosi Albert Luthuli Central Hospital (IALCH), Durban, South Africa, which has been described previously [13,14]. Consecutive patients aged 18 to 60 years who required dialysis and had newly inserted double-cuffed coiled Tenckhoff catheters were recruited between September 2012 and February 2015 stopping enrollment when each cohort had 70 participants. Sixty HIV-positive and 59 HIV-negative patients who had at least one nasal swab sample taken during follow-up were included in this sub-study. The study protocol was approved by the University of KwaZulu-Natal Biomedical Research Ethics Committee (BE 187/11), and informed consent was obtained from the patients before enrollment. The status of HIV infection was determined by two 4th generation HIV enzyme-linked immunosorbent assays performed by the South African National Health Laboratory Service (NHLS) before enrollment; screening for HIV was performed using a HIV Ag/Ab Combo (CHIV) assay (ADVIA Centaur® XP, Siemens Healthcare Diagnostics, Tarrytown, NY) and confirmation was done using HIV Combi and HIV Combi PT assays (Cobas e601, Roche Diagnostics, Mannheim, Germany). Antiretroviral therapy (ART) was left to the discretion of the local clinic.

Y-sets, twin-bag systems, and conventional peritoneal dialysis (PD) solutions (Dianeal® 1.5%, 2.5%, or 4.25% dextrose, icodextrin, or amino acid-based solutions; Baxter Healthcare, Deerfield, IL) were used in all CAPD patients. They generally performed four exchanges per day. All patients received approximately 40 h of practical and theoretical CAPD training in groups and individualized sessions conducted by the same nursing team of two senior nurses working together. A prophylactic intravenous antibiotic was administered to all patients prior to PD catheter insertion. Patients were prescribed 4% chlorhexidine Surgiscrub soap for hand washing and chlorhexidine 0.5% in ethanol 70% solution for hand rubs between hand washing. They were directed to use water and medicated soaps of their choice for exit site care.

### Enrollment and follow-up

The patients’ demographic, clinical, and biochemical data were documented on enrollment. The patients were followed up monthly at a central renal clinic in IALCH for 18 months or until the endpoints of catheter removal and subsequent transfer to hemodialysis or death. At each follow-up assessment, nasal swabs were taken, phlebotomy was performed for biochemical tests, and the details of infective complications and hospital admissions in the intervening periods were recorded on predefined questionnaires. Full blood counts were performed, and the serum concentrations of C-reactive protein (CRP), urea, creatinine, electrolytes, and albumin were measured at NHLS, and the results were periodically retrieved from the IALCH’s electronic results database.

### Microbiology

Swabbing of the anterior nasal vestibules with sterile swabs (Amies Agar Gel-No Charcoal Transport System; Copan Italia SpA, Brescia, Italy) was performed monthly by a research nurse, and the swabs were transported to the laboratory for processing. Colistin-nalidixic agar and mannitol salt agar media were used for the cultures. The CAPD nurse took PD effluent specimens for white blood cell (WBC) counts and culture when the patients’ clinical presentations suggested peritonitis, and they were transported to the NHLS microbiology department in sterile specimen bottles for processing. The PD effluent WBC counts were determined using a 40× microscope objective lens. The culturing was done on chocolate, blood agar, and brain-heart infusion broth. A Vitek® 2 system (bioMérieux, France) was used for identification and antibiotic susceptibility testing of the nasal swab and PD effluent specimens.

### Definitions

A peritonitis episode was defined as a clinical presentation with a cloudy effluent or abdominal pain associated with a PD effluent WBC count of >100 cells/µL or a positive culture. All patients were treated for at least 2 weeks, and they initially received intraperitoneal vancomycin and amikacin empirically, with further therapy modified according to the culture results. Episodes with culture-confirmed *Staphylococcus* growth and the date of presentation, information about whether the patient was treated as an inpatient or outpatient, and the presenting PD WBC counts were included in this analysis. The infection rates were calculated as the total number of infectious episodes with an organism during the follow-up period divided by the dialysis-years’ time at risk, and they were expressed as the number of episodes per year [15].

Exit site infections were diagnosed clinically and defined based on the presence of purulent drainage, with or without skin erythema, at the catheter-epidermal interface. Tunnel infections were diagnosed clinically or using sonographic studies, and they were defined based on the presence of erythema, edema, or tenderness over the subcutaneous pathway [15]. Both infection types were referred to as catheter infections.

The participants were classified as *S. aureus* nasal carriers if at least one culture from the monthly nasal swabs was positive for *S. aureus*, and they were classified as non-carriers if none of the cultures from the monthly nasal swabs was positive for *S. aureus* during follow-up. The *S. aureus* nasal carriers were further classified as intermittent *S. aureus* carriers if only one nasal culture was positive for *S. aureus* during follow-up or as persistent carriers if more than one monthly nasal culture was positive for *S. aureus.*

### Mupirocin exposure

Exposure to mupirocin during the study was determined through evaluation of the electronic hospital database at the end of the study period for instances where mupirocin was dispensed during each patient’s follow-up period. The date of the first documented prescription of mupirocin and the number of months prescribed were recorded for individual patients.

### Statistical analysis

The continuous variables were expressed as the mean ± standard deviation (SD) or median and the interquartile range (IQR), and they were compared using Student’s *t*-test or the Wilcoxon-Mann-Whitney test, as appropriate. The proportions and categorical variables were compared using the *χ*^2^ test or Fisher’s exact test, as appropriate. The Mantel-Haenszel method was used to calculate rate ratios and to compare incidence rates in the two study cohorts and the subgroups divided according to CD4+ cell count and *S. aureus* nasal carriage. Logistic regression was used to assess the relationship between HIV and the detectability of *S. aureus* in the nares. A multivariable logistic regression model that included age, race, gender, smoking, alcohol use, diabetes, body mass index (BMI), baseline serum albumin, baseline CRP level, baseline CD4+ cell count, Tenckhoff catheter insertion parameters (site and method, whether laparoscopic or percutaneous), mupirocin exposure to the nose, type of primary residence (urban vs. rural), highest education level, employment (employed vs. unemployed), number of total peritonitis episodes experienced and total number of hospital days spent as inpatient in hospital during follow-up, was used to determine whether HIV independently predicts the detection of *S. aureus* in the nares.

Cox proportional hazard analysis was used to estimate the associations between HIV infection and the peritonitis outcome events due to *Staphylococcus* species, *S. aureus,* and CNS, respectively. Multivariable Cox proportional hazard analysis was used to identify independent predictors of each *Staphylococcal* peritonitis event type. The covariates included in the all Cox models for the peritonitis outcome variables were age, race, gender, smoking, diabetes, BMI, baseline serum albumin, baseline CD4+ cell count, Tenckhoff catheter insertion parameters (site and method), mupirocin exposure, type of primary residence, highest education level, and employment. In the Cox model for *Staphylococcus* species peritonitis, further covariates of *Staphylococcus* species nasal carriage and catheter infection were added. In the Cox model for *S. aureus* peritonitis, additional covariates of the *S. aureus* nasal carriage and catheter infection were included. In the Cox model for CNS peritonitis, additional covariates of CNS nasal carriage and *Staphylococcus* species catheter infection were included. All the analyses were performed using Stata, version 15.0 (StataCorp LP, College Station, TX), and the significance level was set at *p <* 0.05.

## Results

### Patients’ characteristics

The study population of 119 CAPD patients included 59 HIV-negative and 60 HIV-positive patients with a median age of 39 years (IQR: 29–49 years) and 34 years (IQR: 30–41.5 years), respectively, (*p =* 0.207). Fifty-two percent of the HIV-positive patients were either newly diagnosed with HIV or had recently been started on ART, less than six months before Tenckhoff catheter insertion. Sixty percent of the HIV-positive patients had a suppressed viral load of <150 copies/mL, which was the hospital laboratory assay’s limit at the time of enrollment. While the median baseline viral load was 4 229.5 copies/mL (IQR: 817–88,294.5 copies/mL) for the patients with detectable viral loads, the median fell below the detectable limit (IQR: <150–2284.5 copies/mL) when the patients with undetectable viral loads were included. The characteristics of the study population are outlined in Table 1.

**Table 1.** Baseline characteristics of the patients.

	HIV negative (*n* = 59)	HIV positive (*n* = 60)	*p* Value
Mean ± SD age (years)	38.8 ± 11.6	36.2 ± 9.2	0.168^a^
Mean ± SD weight (kg)	68.6 ± 12.3	65.4 ± 13.6	0.181^a^
Body mass index, median (IQR)	23.8 (21.8–28.4)	22.8 (20.7–27.9)	0.239^b^
Mean ± SD waist circumference (cm)	90.4 ± 10.4	89.6 ± 11.5	0.703^a^
Sex			
Female, *n* (%)	24 (40.7)	35 (58.3)	0.054^c^
Race			
African, *n* (%)	50 (84.8)	60 (100.0)	0.001^d^
Indian, *n* (%)	7 (11.9)	0 (0.0)	
Mixed race, *n* (%)	2 (3.4)	0 (0.0)	
Hypertension, *n* (%)	54 (91.5)	52 (75.0)	0.026^d^
Diabetes, *n* (%)	3 (5.1)	6 (10.0)	0.491^d^
SLE, *n* (%)	3 (5.1)	1 (1.7)	0.364^d^
Hepatitis B, *n* (%)	6 (10.2)	6 (10.0)	0.974^c^
Primary residence			
Rural, *n* (%)	20 (33.9)	21 (35)	0.847^c^
Urban, *n* (%)	39 (66.1)	38 (63.3)	
Education level			
Primary school, *n* (%)	15 (25.4)	11 (18.6)	0.649^c^
High school, *n* (%)	26 (44.1)	27 (45.8)	
Post-grade 12, *n* (%)	18 (30.5)	21 (35.6)	
Employment status			
Unemployed, *n* (%)	43 (72.88)	47 (78.3)	0.387^c^
Employed, *n* (%)	16 (27.1)	12 (20.0)	
Tenckhoff catheter insertion method			
Laparoscopic, *n* (%)	57 (96.6)	31 (51.7)	<0.001^d^
Percutaneous, *n* (%)	2 (3.4)	29 (48.3)	
Haemoglobin (g/dL), median (IQR)	9.45 (8.2–11.2)	8.95 (7.8–9.8)	0.038^b^
Mean ± SD albumin, g/L	35.5 ± 6.8	31.06 ± 6.8	0.002^a^
eGFR (mL/min/1.73 m^2^), median (IQR)	6 (4–9)	6 (5–8)	0.940^b^
Creatinine (µmol/L), median (IQR)	728 (529–1004)	710.5 (592–880)	0.941^b^
CRP (mg/L), median (IQR)	18 (6–34)	48.5 (18.5–102.5)	<0.001^b^
ESR (mm/hr), median (IQR)	48 (29–61)	88 (50–129)	<0.001^b^
Ferritin (µg/L), median (IQR)	626 (335–1047)	565 (378.5–905.5)	0.770^b^
CD4^+^ cell count			
Mean ± SD cells/µL		407.8 ± 238.6	
CD4^+^ <200 cells/µL, *n* (%)		9 (15.0)	
CD4^+^ 200–350 cells/µL, *n* (%)		18 (30.0)	
CD4^+^ 350–500 cells/µL, *n* (%)		18 (30.0)	
CD4^+^ ≥500 cells/µL, *n* (%)		15 (25.0)	
Viral load			
Median, copies/mL (IQR)		4 229.5 (817–88 294.5)	
Suppressed (<150 copies/mL), *n* (%)		36 (60.0)	
150–1000 copies/mL, *n* (%)		7 (11.7)	
>1000 copies/mL, *n* (%)		17 (28.3)	
ART history at enrollment			
<6 months, *n* (%)		31 (51.7)	
6–12 months, *n* (%)		8 (13.3)	
>1 year, *n* (%)		21 (35.0)	
ART drug regimen			
3TC/EFV/ABC, *n* (%)		50 (83.3)	
3TC/EFV/AZT, *n* (%)		2 (3.3)	
3TC/EFV/D4T, *n* (%)		3 (5.0)	
3TC/NVP/ABC, *n* (%)		3 (5.0)	
Alluvia/ABC/3TC, *n* (%)		1 (1.7)	
AZT/3TC/aluvia, *n* (%)		1 (1.7)	

a *t*-test for comparison of means.

b Wilcoxon-Mann-Whitney test.

c Pearson’s *χ*^2^ test.

d Fisher’s exact test.

SD: standard deviation; IQR: interquartile range; HIV: human immunodeficiency virus; CD: cluster of differentiation; SLE: systemic lupus erythematosus; eGFR: estimated glomerular filtration rate (Modification of Diet in Renal Disease equation); ART: highly active antiretroviral therapy; ESR: erythrocyte sedimentation rate; 3TC: lamivudine; EFV: efavirenz; ABC: abacavir; AZT: zidovudine; D4T: stavudine; NVP: nevirapine; CRP: C-reactive protein.

### Study drop out

After 18 months, 64.4% (38 of 59) of the HIV-negative patients and 33.3% (20 of 60) of the HIV-positive patients were alive with patent catheters (*p =* 0.001). Twenty-two percent (13 of 59) of the HIV-negative patients and 25.0% (15 of 60) of the HIV-positive patients (*p =* 0.703) had their Tenckhoff catheters removed because of malfunctions or infective complications, and 10.2% (6 of 59) of the HIV-negative patients and 36.7% (22 of 60) of the HIV-positive patients had died (*p =* 0.001). Forty-eight percent (13 of 27) of HIV-positive patients with baseline CD4 counts <350 cells/µL died, and 27.3% (9/33) of HIV-positive patients with CD4 counts ≥350 cells/µL died, both significantly higher than the HIV-negative proportion (*p <* 0.001). Two HIV-negative and three HIV-positive patients were lost to follow-up due to live related renal transplantation, improved renal functions, or opted for private hemodialysis (Supporting Information Table 1).

### Staphylococcal nasal carriage

Thirty percent (18 of 59) of the HIV-negative patients and 43.3% (26 of 60) of the HIV-positive patients had detectable *S. aureus* in the nares (*p =* 0.147) (Table 3). On univariate logistic regression, HIV was not significantly associated with the detection of *S. aureus* in the nares (Odds Ratios [OR] 1.74, 95% confidence interval [CI] 0.82–3.70, *p =* 0.149). However, on multivariable logistic regression HIV (adjusted OR 3.91, CI 1.07–14.35, *p =* 0.040) and baseline CD4+ count were found to be independent predictors for *S. aureus* detection in the nares (Table 2). Methicillin-resistant *S. aureus* (MRSA) was found in 13.6% (8 of 59) of the HIV-negative patients and 31.7% (19 of 60) of the HIV-positive patients (*p =* 0.018). The median time from Tenckhoff catheter insertion to the first MRSA detection was 140.5 days (IQR: 97–317.5 days) for HIV-negative patients and 63 days (IQR: 31–80 days) for HIV-positive patients (*p* = 0.008) (Table 3). On logistic regression HIV was associated with a significant OR of 2.95 (95% CI 1.17–7.43, *p =* 0.021) for the detection of MRSA in the nares, and after adjustments HIV remained an independent predictor for the detection of MRSA in the nares with an adjusted OR of 7.35 (95% CI 1.45–37.35, *p =* 0.016) (Table 2). Coagulase-negative staphylococci were detectable in the nares of 69.5% (41 of 59) in the HIV-negative cohort and 43.3% (26/60) in the HIV-positive cohort (*p =* 0.004) (Table 3).

**Table 2.** Univariate and multivariable logistic regression analyses: Risk factors vs. *Staphylococcus aureus* nasal colonization.

	Univariate logistic regression analysis		Multivariable logistic regression analysis^a^	
Variable	OR (95% CI)	*p* Value	OR (95% CI)	*p* Value
*Staphylococcus aureus* nasal carriage				
HIV	1.74 (0.82–3.70)	0.149	3.91 (1.07–14.35)	0.040
Baseline albumin	0.95 (0.90–1.01)	0.086	0.95 (0.88–1.02)	0.134
Baseline CD4+ cell count (cells/µL)				
HIV–negative	Reference			
CD4+< 350	2.23 (0.71–6.97)	0.167	0.24 (0.06–0.91)	0.036
CD4+≥ 350	3.64 (1.30–10.19)	0.014	1.00	
MRSA nasal carriage				
HIV	2.95 (1.17–7.43)	0.021	7.35 (1.45–37.35)	0.016
Baseline albumin	0.9 (0.84–0.96)	0.002	0.88 (0.8–0.97)	0.007
Baseline CD4+ cell count (cells/µL)				
HIV–negative	Reference			
CD4+<350	2.23 (0.71–6.97)	0.167	0.22 (0.05–0.95)	0.042
CD4+≥350	3.64 (1.3–10.19)	0.014	1.00	

a Multivariable logistic regression model for *Staphylococcus aureus* and MRSA nasal colonization outcomes included variables of HIV, age, race, gender, smoking, alcohol use, diabetes, body mass index (BMI), baseline serum albumin, baseline CRP level, baseline CD4+ cell count, Tenckhoff catheter insertion parameters (site and method, whether laparoscopic or percutaneous), mupirocin exposure to the nose, type of primary residence (urban vs. rural), highest education level, employment (employed vs. unemployed) and number of total peritonitis episodes experienced and total number of hospital days spent as inpatient in hospital during follow-up.

CI: confidence interval; HIV: human immunodeficiency virus; CD: cluster of differentiation; OR: Odds Ratio.

**Table 3.** Staphylococcal nasal colonization rates according to HIV seropositive status.

	HIV negative	HIV positive	*p* Value
*Staphylococcus aureus* nasal carriage	30.5% (18/59)	43.3% (26/60)	0.147^a^
*Staphylococcus aureus* nasal carriage among HIV-positive patients with:			
CD4+ count <350 cells/µL		33.3% (9/27)	0.793^a^
CD4+ count ≥350 cells/µL		51.5% (17/33)	0.047^a^
Intermittent *S. aureus* carrier	10.2% (6/59)	10.0% (6/60)	0.976^a^
Persistent *S. aureus* carrier	20.3% (12/59)	33.3% (20/60)	0.110^a^
Time to first *S. aureus* nasal detection (days), median (IQR)	251 (97–377)	67.5 (41–131)	0.002^b^
<30 days (prevalent cases)	0	8.3% (5/60)	0.035^c^
30–180 days	13.6% (8/59)	26.7% (16/60)	
180–365 days	8.5% (5/59)	5.0% (3/60)	
12–18 months	8.5% (5/59)	3.3% (2/60)	
MRSA nasal carriage	13.6% (8/59)	31.7% (19/60)	0.018^a^
MRSA nasal carriage among HIV-positive patients with:			
CD4+ count <350 cells/µL		25.9% (7/27)	0.161^a^
CD4+ count ≥350 cells/µL		36.4% (12/33)	0.011^a^
Time to first MRSA nasal detection (days), median (IQR)	140.5 (97–317.5)	63 (31–80)	0.008^b^
<30 days (prevalent cases)	0	6.7% (4/60)	0.007^c^
30–180 days	8.5% (5/59)	23.3% (14/60)	
180–365 days	1.7% (1/59)	1.7% (1/60)	
12–18 months	3.4% (2/59)	0	
Coagulase-negative staphylococcal nasal carriage	69.5% (41/59)	43.3% (26/60)	0.004^a^
*Staphylococcus* species nasal carriage	86.4% (51/59)	75.0% (45/60)	0.114^a^

a Pearson’s *χ*^2^ test.

b Wilcoxon rank-sum (Mann-Whitney) test.

c Fisher’s exact test.

HIV: human immunodeficiency virus; IQR: interquartile range; MRSA: methicillin-resistant *Staphylococcus aureus*.

### Staphylococcal peritonitis

*Staphylococcus* species (spp) was cultured from 16 HIV-negative and 29 HIV-positive peritonitis episodes that occurred in 17.0% (10 of 59) of the HIV-negative and 28.3% (17 of 60) of the HIV-positive patients. Forty-four percent (7 of 16) of these peritonitis episodes cultured methicillin-sensitive *S. aureus* in the HIV-negative cohort and 10.3% (3 of 29) in the HIV-positive cohort (*p =* 0.021) (Supporting Information Table 1). The HIV-positive cohort was associated with a higher staphylococcal peritonitis rate compared with the HIV-negative cohort (0.569 vs. 0.223 episodes/person-years; rate ratio [RR] 2.55, 95% CI 1.36–4.77, *p =* 0.002) (Table 4) (Supporting Information Figure 1). HIV was associated with higher staphylococcal peritonitis events censored for death, technique failure and loss to follow-up on both univariate (hazard ratio [HR] 2.58. 95% CI 1.37–4.85, *p =* 0.003) and multivariable (adjusted HR [AHR] 2.85, 95% CI 1.19–6.84, *p =* 0.019) Cox proportional hazard analysis (Tables 4 and 5).

**Table 4.** Incidence rates of staphylococcal infections.

	HIV negative (episodes/person-years)	HIV positive (episodes/person-years)	RR (95% CI)	*p* Value
Staphylococcal peritonitis	0.223	0.569	2.55 (1.36–4.77)	0.002
CD4+ <200 cells/µL		0.973	4.36 (1.45–13.15)	0.004^a^
CD4+ ≥200 cells/µL		0.532	2.38 (1.25–4.55)	0.006^a^
*S. aureus* peritonitis	0.129	0.136	1.05 (0.39–2.82)	0.920
CD4+ <200 cells/µL		0.877	6.79 (1.84–25.09)	0.001^a^
CD4+ ≥200 cells/µL		0.083	0.64 (0.20–2.09)	0.460^a^
*S. aureus* nasal carriers	0.284	0.256	0.90 (0.30–2.68)	0.853
			6.38 (1.32–30.71)	0.008^b^
Non-carriers	0.044	0.036	0.80 (0.07–8.84)	0.856
			7.18 (0.86–59.65)	0.033^c^
CNS peritonitis	0.089	0.401	4.53 (1.83–11.22)	0.0003
CD4+ <200 cells/µL		0.205	2.31 (0.28–19.22)	0.424^a^
CD4+ ≥200 cells/µL		0.421	4.76 (1.91–11.84)	0.0002^a^
CNS nasal carriers	0.109	0.504	4.62 (1.66–12.81)	0.001
			2.40 (0.28–20.55)	0.409^d^
Non-CNS carriers	0.046	0.285	6.25 (0.77–50.81)	0.0495
			1.77 (0.72–4.39)	0.210^e^
All-cause catheter infection	0.160	0.286	1.78 (0.79–4.02)	0.156
*S. aureus* catheter infection	0.076	0.156	2.06 (0.67–6.30)	0.195
*S. aureus* nasal carriers	0.090	0.302	3.34 (0.69–16.09)	0.110
			1.32 (0.22–7.90)	0.760^b^
Non*-*carriers	0.068	0.036	0.52 (0.05–5.00)	0.565
			8.48 (1.04–68.91)	0.016^c^

a HIV-negative group used as the reference group.

b *Staphylococcus aureus* nasal carriers versus non-carriers in the HIV-negative cohort.

c *Staphylococcus aureus* nasal carriers versus non-carriers in the HIV-positive cohort.

d Coagulase-negative staphylococci nasal carriers versus non-carriers in the HIV-negative cohort.

e Coagulase-negative staphylococci nasal carriers versus non-carriers in the HIV-positive cohort.

CI: confidence interval; HIV: human immunodeficiency virus; CD: cluster of differentiation; RR: rate ratio; CNS: coagulase-negative staphylococci.

**Table 5.** Cox proportional hazard univariate and multivariable analyses: risk factors versis *Staphylococcal peritonitis*.

	Univariate Cox proportional hazards		Multivariable Cox proportional hazards	
Variable	HR (95%CI)	*p* Value	HR (95% CI)	*p* Value
Staphylococci species peritonitis^a^				
	*n* = 119		*n* = 118^b^	
HIV	2.58 (1.37–4.85)	0.003	2.85 (1.19–6.84)	0.019
Diabetes	2.80 (1.25–6.30)	0.013	5.04 (1.60–15.87)	0.006
*Staphylococcus* species nasal carriage	2.83 (0.68–11.75)	0.152	3.05 (0.68–13.72)	0.147
Body mass index	0.94 (0.87–1.01)	0.108	0.95 (0.87–1.04)	0.234
Baseline serum albumin	0.96 (0.91–1.00)	0.062	0.97 (0.91–1.02)	0.239
Mupirocin ointment exit-site exposure	1.13 (0.54–2.36)	0.738	2.01 (0.74–5.50)	0.173
Coagulase-negative staphylococci peritonitis^c^				
	*n* = 119		*n* = 118^b^	
HIV	4.80 (1.93–11.94)	0.001	7.64 (2.18–26.82)	0.001
Diabetes	1.92 (0.58–6.39)	0.286	3.90 (0.75–20.25)	0.105
CNS nasal carriage	1.45 (0.64–3.32)	0.376	1.66 (0.63–4.39)	0.309
Body mass index	0.90 (0.81–1.00)	0.045	0.85 (0.76–0.96)	0.006
Baseline serum albumin	0.95 (0.90–1.02)	0.142	0.93 (0.86–1.02)	0.109
*Staphylococcus aureus* peritonitis^d^				
	*n* = 119		*n* = 118^b^	
HIV	0.96 (0.36–2.60)	0.942	1.18 (0.25–5.63)	0.833
Gender				
female	Reference			
male	3.11 (1.00–9.65)	0.049	4.45 (1.12–17.67)	0.034
Diabetes	5.54 (1.77–17.30)	0.003	12.98 (1.92–87.81)	0.009
*S. aureus* nasal carriage	6.90 (1.97–24.25)	0.003	15.20 (3.01–76.75)	0.001
Body mass index	0.99 (0.90–1.10)	0.923	1.02 (0.86–1.21)	0.800
Baseline serum albumin	0.97 (0.90–1.04)	0.340	1.02 (0.90–1.16)	0.719
Baseline CD4 count				
HIV-negative	Reference			
CD4 < 200	4.49 (1.19–17.01)	0.027	27.30 (2.40–310.27)	0.008
CD4 > = 200	0.61 (0.19–2.00)	0.416	1.00	

a Cox model for Staphylococci species peritonitis outcome included variables of HIV, age, race, gender, smoking, diabetes, body mass index, baseline serum albumin, primary residence, highest education level, employment, baseline CD4 count, Tenckhoff catheter insertion site, Tenckhoff catheter insertion method (laparoscopic vs. percutaneous), staphylococci species nasal carriage, staphylococci species catheter infection, and exposure to topical mupirocin at exit site.

b One patient record excluded in analysis due to missing data on primary residence, education, and employment.

c Cox model for coagulase-negative staphylococci peritonitis outcome included variables of HIV, age, race, gender, smoking, diabetes, body mass index, baseline serum albumin, primary residence, highest education level, employment, baseline CD4 count, Tenckhoff catheter insertion site, Tenckhoff catheter insertion method (laparoscopic vs. percutaneous), coagulase-negative staphylococci nasal carriage, staphylococci catheter infection, and exposure to topical mupirocin at exit site.

d Cox model for *Staphylococcus aureus* peritonitis outcome included variables of HIV, age, race, gender, smoking, diabetes, body mass index, baseline serum albumin, primary residence, highest education level, employment, baseline CD4 count, Tenckhoff catheter insertion site, Tenckhoff catheter insertion method (laparoscopic vs. percutaneous), *Staphylococcus aureus* nasal carriage, *Staphylococcus aureus* catheter infection, and exposure to topical mupirocin at exit site.

BMI: body mass index; CI: confidence interval; CD: cluster of differentiation; HR: hazard ratio; HIV: human immunodeficiency virus.

Coagulase-negative staphylococci peritonitis rates were 0.089 episodes/person-years in the HIV-negative cohort and 0.401 episodes/person-years in the HIV-positive cohort (RR 4.53, CI 1.83–11.22, *p =* 0.0003). On multivariable Cox proportional hazard analysis, HIV (AHR 7.64, CI 2.18–26.82, *p =* 0.001), and BMI were found to be independent predictors of CNS peritonitis.

*S. aureus* peritonitis rates were 0.129 episodes/person-years in the HIV-negative cohort and 0.136 episodes/person-years in the HIV-positive cohort (RR 1.05, CI 0.39–2.82, *p =* 0.920). HIV was not significantly associated with *S. aureus* peritonitis events on both univariate (HR 0.96, CI 0.36–2.60, *p =* 0.942) and multivariable Cox proportional hazard analysis (AHR 1.18, CI 0.25–5.63, *p =* 0.833). However, HIV-positive patients with CD4+ cell count <200 cells/µL had a *S. aureus* peritonitis rate of 0.877 episodes/person-years that was much higher than the rate of the HIV-negative cohort (RR 6.79, CI 1.84–25.09, *p =* 0.001). On multivariable analysis, a CD4+ cell count <200 cells/µL (AHR 27.30, CI 2.40–310.27, *p =* 0.008), male gender, *S. aureus* nasal carriage, and diabetes were found to be independent predictors of *S. aureus* peritonitis.

### Exit site and tunnel infections

Ten catheter infection episodes occurred in the HIV-negative cohort, and 14 catheter infection episodes occurred in the HIV-positive cohort in 15.2% (9 of 59) and 18.3% (11 of 60) of the patients, respectively. *S. aureus* was cultured from the exit site pus swabs or tunnel abscess aspirates in five HIV-negative and eight HIV-positive catheter infection episodes. The *S. aureus* catheter infection rates were 0.076 episodes/person-years in the HIV-negative cohort and 0.156 episodes/person-years in the HIV-positive cohort (RR 2.06, CI 0.67–6.30, *p =* 0.195) (Table 4). The *S. aureus* catheter infection rates were 0.199 episodes/person-years for the *S. aureus* nasal carriers and 0.056 episodes/person-years for the non-carriers (RR 3.57, CI 1.10–11.59, *p =* 0.024).

### Mupirocin exposure

Eighty-six percent (51 of 59) of the HIV-negative cohort had mupirocin ointment prescribed for exit site application compared to 60.0% (36 of 60) of the HIV-positive cohort (*p =* 0.001), due to the earlier recruitment of a greater proportion of HIV-negative patients when hospital policy favored routine mupirocin exit site prophylaxis. Furthermore, mupirocin ointment was prescribed for a median 5 (2–9) months in the HIV-negative cohort compared to 3 (2–6.5) months in the HIV-positive cohort (*p =* 0.140). Fourteen percent (8 of 59) of the HIV-negative cohort had mupirocin nasal spray prescribed during follow-up compared to 15.0% (9 of 60) in the HIV-positive cohort (*p =* 0.822).

## Discussion

This prospective cohort study evaluated the effects of HIV infection on the *S. aureus* nasal carriage and CAPD-associated staphylococcal infective outcomes in patients with ESRD who required dialysis. HIV infection was associated with significantly higher MRSA nasal carriage and staphylococcal peritonitis rates, and HIV-positive patients with CD4 count ≥350 cells/µL had significantly higher *S. aureus* nasal colonization rates. However, our study failed to demonstrate any significant differences with respect to catheter infection rates in relation to HIV infection. CD4+ cell count <200 cells/µL in the HIV-positive cohort and *S. aureus* nasal carriage, were found to independently predict *S. aureus* peritonitis.

The difference between the HIV-positive cohort and HIV-negative cohort in relation to the *S. aureus* nasal carriage rate was not statistically significant, but HIV-positive patients with baseline CD4+ count ≥350 cells/µL were associated with significantly higher *S. aureus* nasal colonization compared to the HIV-negative cohort. Furthermore, HIV and baseline CD4+ cell counts were found to independently predict the detection of nasal *S. aureus* in our multivariable logistic regression model, and *S. aureus* nasal carriage was detected significantly earlier during follow-up in the HIV-positive cohort compared to the HIV-negative cohort. *S. aureus* nasal colonization rates were lower in the HIV-positive patients with CD4+ counts <350 cells/µL compared to those with CD4+ counts ≥350 cells/µL. This observation may have been influenced by the increased dropout rate in the HIV-positive cohort attributed to death, which predominantly affected the HIV-positive subgroup with CD4+ counts <350. The decreased observation times may have undermined the detection of *S. aureus* in this subgroup compared to longer observation times among those with higher CD4+ counts and those in the HIV-negative cohort. These results suggest an association of HIV with *S. aureus* nasal colonization that may be better defined in a larger more adequately powered study.

The MRSA nasal carriage rate in the HIV-positive cohort was significantly higher than that in the HIV-negative cohort, and that it was much higher than the pooled MRSA nasal carriage rate estimate of 6.9% (95% CI 4.8–9.3) reported in a meta-analysis of HIV-positive non-CAPD populations [16]. Furthermore, HIV positive status and baseline CD4+ cell count were significant factors associated with MRSA nasal colonization on both univariate and multivariable logistic regression analysis. These significant relations highlight the increased risk of MRSA colonies in the nares associated with HIV infection, which raises concerns about the subsequent development of more serious MRSA infections. Furthermore, the significantly higher proportion of episodes of methicillin-sensitive *S. aureus* peritonitis in the HIV-negative cohort than in the HIV-positive cohort highlights the relatively low burden of methicillin-resistant infections in the HIV-negative group. Infection with HIV has been positively linked to an increased risk of MRSA colonization and subsequent infection in the general population [17–19]. Colonization and infection by MRSA, which is commonly acquired through nosocomial contact, have been associated with exposure to antibiotics, prior hospitalization, illicit drug use, chronic skin disease, and risky lifestyle behaviors in the general population [18,20]. Healthcare- and antibiotic-associated exposures may have increased the risk of MRSA colonization in our HIV-positive CAPD cohort; however, these variables were not directly measured or controlled for in our study design. The community-associated acquisition could also have contributed to the earlier detection of MRSA colonization in our HIV-positive cohort, as compared to the later detection of MRSA colonization in the HIV-negative cohort, suggesting a more traditional nosocomial-associated acquisition in the HIV-negative cohort [21].

The HIV-positive cohort had a higher staphylococcal peritonitis rate than the HIV-negative cohort, because of the significantly increased CNS peritonitis incidence in the former compared with that in the latter, which highlights the increased vulnerability of HIV-positive patients to touch contamination. Coagulase-negative staphylococci is a common skin commensal found in many parts of the body (nose, axilla, groin, etc.) to various degrees [22,23]. It is also the most commonly isolated pathogen causing peritonitis in patients on CAPD [24]. Factors associated with the development of CNS peritonitis are access to the peritoneum via the catheter, bacterial characteristics allowing evasion of host defenses, immune depression induced by conventional PD fluids, and inherent host immune system dysfunction [22,25,26]. In this study, HIV was found to increase the risk of developing CNS peritonitis, reflecting the immunosuppressive state of HIV and the resultant impaired ability of local peritoneal immune defense mechanisms to combat the contaminating CNS organisms. Furthermore, CNS nasal carriage was found to be significantly increased in the HIV-negative cohort compared to that in the HIV-positive cohort, suggesting HIV-associated changes to the typical body commensal patterns favoring organisms such as MRSA, which are associated with greater healthcare exposure. However, CNS nasal carriage was not significantly associated with CNS peritonitis. Previous reports have also shown a disconnect between CNS strains colonizing the body and those causing infection, as peritonitis-cultured strains tended to differ from those isolated from other body sites before infection [22,23].

On multivariable analysis, HIV and diabetes were prominent independent predictors for the development of staphylococcal peritonitis, reinforcing the suggested risks attributed to impaired immunity. Furthermore, HIV and BMI were prominent independent predictors for the development of CNS peritonitis. BMI was found to be a protective predictor for this type of peritonitis. A result also reported by another South African study by Isla et al. [27] where BMI was protective for all-cause peritonitis. These results may suggest a nutritional protective effect, in contrast to some peritonitis publications that have reported an increased peritonitis risk associated with higher BMI [28–30]. This adverse risk associated with lower BMI probably reflects increased hazards due to undernutrition and the resultant immunocompromise hindering effective containment of advancing CNS organisms contaminating the CAPD system. Both these South African studies were likely influenced by a national rationing policy favoring ESRD patients with lower BMI levels (<35 kg/m^2^) for access to scarce renal replacement therapy, thus limiting the ability to detect possible hazards associated with obesity [31].

The *S. aureus* peritonitis rate was not affected by HIV infection status, as reflected by similar peritonitis rates between the two cohorts. However, HIV-positive patients with CD4+ counts <200 cells/µL were noted to have a six-fold higher *S. aureus* peritonitis rate compared to the HIV-negative cohort. Moreover, this HIV-positive sub-group was associated with increased hazards for developing *S. aureus* peritonitis events both on univariate and multivariable Cox proportional hazard models suggesting a peritonitis risk profile influenced by changes in immunological state and likely resulting from impaired local immunity [25]. Further, on multivariable analysis, diabetes and *S. aureus* nasal carriage were found to be other independent predictors of *S. aureus* peritonitis. These associations support a role for decreased immunity and *S. aureus* nasal carriage in the risk profile for *S. aureus* peritonitis.

Compared with the HIV-negative state, HIV infection was associated with higher *S. aureus* and all-cause catheter infection rates. However, these differences were not statistically significant, which was probably a consequence of the lower numbers of these outcomes. The study’s protocol did not restrict the use of mupirocin prophylaxis, either at the exit sites or the nares for ethical reasons, because these prophylactic measures significantly reduce *S. aureus*-associated catheter infections [32]. However, mupirocin was not uniformly used by treating physicians. Concerns of resistance led to mupirocin being withdrawn from use in general CAPD by the hospital’s therapeutics committee midway through the study period, thereafter reserved for nasal decolonization of *S. aureus*. Nevertheless, the sporadic use of mupirocin likely suppressed the incidence of exit site and tunnel infections.

The main limitation of our study is that it was a single-center observational study, which limits the causation inferences that can be drawn. The sample size may have been too small for differences in the *S. aureus* nasal colonization and catheter infection outcomes to be fully appreciated and may have led to wide confidence intervals in some observed associations. The reported outcomes were secondary outcomes in the parent study and were not the primary determinants of the sample size calculations. Furthermore, the disproportionately higher mortality rate in the HIV-positive cohort contributed to a significantly higher dropout rate and a significantly shorter observation time compared to the HIV-negative cohort. This observation-time bias may have resulted in an underestimation of *S. aureus* nasal colonization and peritonitis, and catheter-associated infection rates in the HIV-positive cohort. However, it is not expected to have meaningfully altered observed associations, such as the CNS peritonitis risk associated with HIV, as this kind of potential bias is likely to have led to an underestimation of observed associations rather than enhance them. The differences in the *S. aureus* nasal colonization and infection rates require further investigation with additional research in prophylactic measures.

This study’s findings suggest that HIV infection adversely influences MRSA nasal colonization and that it may increase the risk of CNS peritonitis. Differences in the *S. aureus* peritonitis and catheter infection rates in relation to HIV infection were not significant. However, *S. aureus* nasal carriage and a CD4+ cell count <200 cells/µL associated with HIV were shown to adversely influence the risk for *S. aureus* peritonitis. These observations contribute to our understanding of the resistance profiles of *S. aureus* colonizers and the staphylococcal organism patterns that are likely to cause infection, which may assist in guiding appropriate antibiotic therapy and prophylaxis.

## Supplementary Material

Supplementary Table
