# Supplementary material for: Association of Staphylococcus nasal colonization and HIV in end-stage renal failure patients undergoing peritoneal dialysis
Source: Ren Fail. 2019 Apr 17;41(1):303–13. doi: 10.1080/0886022X.2019.1598433 (PMC6484474; doi:10.1080/0886022X.2019.1598433)
Supplement: Supplementary Table [file IRNF_A_1598433_SM1469.docx]

SUPPLEMENTARY TABLE 1: Patient Outcomes at 18 Months

|  | **HIV negative** | **HIV positive** | ***p* value** |
| --- | --- | --- | --- |
| **Alive with patent catheter at 18 months** | 64.4% (38/59) | 33.3% (20/60) | 0.001^a^ |
| **Mortality** | 10.2% (6/59) | 36.7% (22/60) | 0.001^a^ |
| CD4 count < 350 cells/µL |  | 21.7% (13/60) |  |
| CD4 count ≥ 350 cells/µL |  | 15% (9/60) |  |
| ***Mortality causes*** |  |  |  |
| Bacterial peritonitis and sepsis | 1.7% (1/59) | 5.0% (3/60) |  |
| Sepsis without peritonitis | 0 | 6.7% (4/60) |  |
| Fungal peritonitis | 0 | 1.7% (1/60) |  |
| Cardiovascular Death | 0 | 5.0% (3/60) |  |
| Home Death (unknown cause) | 8.5% (5/59) | 16.7% (10/60) |  |
| Other | 0 | 1.7% (1/60) |  |
| **Catheter failure** | 22.0% (13/59) | 25.0% (15/60) | 0.703^a^ |
| ***Technique failure causes*** |  |  |  |
| Bacterial peritonitis | 13.6% (8/59) | 11.7% (7/60) |  |
| Fungal peritonitis | 3.4% (2/59) | 6.7% (4/60) |  |
| TB Peritonitis | 0 | 1.7% (1/60) |  |
| Blocked catheter/Peritoneal sclerosis | 5.1% (3/59) | 5.0% (3/60) |  |
| **Lost** | 3.4% (2/59) | 5.0% (3/60) | 1.00^b^ |
| Renal transplant | 1.7% (1/59) | 0 |  |
| Improved renal function | 1.7% (1/59) | 3.3% (2/60) |  |
| Patient initiated transfer to Hemodialysis | 0 | 1.7% (1/60) |  |
|  |  |  |  |
| **Mupirocin exit site exposure** | 86.4% (51/59) | 60.0% (36/60) | 0.001 |
| Mupirocin exit site prescribed months, median (IQR) | 5 (2–9) | 3 (2–6.5) | 0.140 |
| **Mupirocin nasal exposure** | 13.6% (8/59) | 15.0% (9/60) | 0.822 |
| Mupirocin nasal prescribed months, median (IQR) | 1 (1–2) | 1 (1–3) | 0.472 |
|  |  |  |  |
| **Staphylococcal peritonitis episodes**, *n* (*n* excluding relapse^d^)  Cultured microorganisms | 16 (15) | 29 (28) | 0.046^a^ |
| MSSA | 43.8% (7/16) | 10.3% (3/29) | 0.021^b^ |
| MRSA | 12.5% (2/16) | 13.8% (4/29) | 1.000^b^ |
| MSCNS | 37.5% (6/16) | 48.3% (14/29) | 0.486^a^ |
| MRCNS | 6.3%(1/16) | 27.6% (8/29) | 0.127^b^ |
| Time to peritonitis episode (days), median (IQR) | 213 (67–498.5) | 214 (102–357) | 0.776^c^ |
| PD WBC count (cells/µL), median (IQR) | 734 (256–2,500) | 988 (430–3,420) | 0.366^c^ |
| Outpatient treatment | 56.2% (9/16) | 58.6% (17/29) | 0.878^a^ |
| Inpatient treatment | 43.8% (7/16) | 41.4% (12/29) |  |
| Inpatient stay (days), median (IQR) | 10 (4–12) | 10 (7–22) | 0.419^c^ |
| Episode outcome |  |  |  |
| Continuation of PD | 93.8% (15/16) | 96.6% (28/29) | 0.590^b^ |
| Catheter removal | 6.2% (1/16) | 0 |  |
| Mortality | 0 | 3.4% (1/29) |  |
|  |  |  |  |
| **Culture negative peritonitis episodes**, *n* (*n* excluding relapse^e^) | 10 (9) | 27 (24) | 0.009^a^ |
| Time to peritonitis episode (days), median (IQR) | 212 (106 – 370) | 143 (49 – 323) | 0.432^c^ |
|  |  |  |  |
| **All-cause catheter infection**, *n* episodes (*n* patients) | 10 (9) | 14 (11) | 0.653^a^ |
| Time to all-cause catheter infection (days), median (IQR) | 112.5 (55–244) | 180.5 (48–399) | 0.429^c^ |
| Exit site infection, *n* episodes (*n* patients) | 9 (9) | 10 (8) | 0.358^b^ |
| Tunnel infections, *n* episodes (*n* patients) | 1 (1) | 4 (3) |  |
| ***S. aureus* catheter infection**, *n* episodes (*n* patients) | 5 (4) | 8(7) | 1.000^b^ |
| Time to *S. aureus* catheter infection (days), median (IQR) | 55 (41–107) | 233 (93.5–427.5) | 0.092 |
| *S. aureus* exit site infection, *n* episodes (*n* patients) | 4 (4) | 5 (4) | 1.000^b^ |
| *S. aureus* tunnel infections, *n* episodes (*n* patients) | 1 (1) | 3 (2) |  |

HIV = human immunodeficiency virus; IQR = interquartile range; MSSA = methicillin-sensitive *Staphylococcus aureus*; MRSA = methicillin-resistant *Staphylococcus aureus*; MSCNS = methicillin-sensitive coagulase-negative staphylococci; MRCNS = methicillin-resistant coagulase-negative staphylococci; PD = peritoneal dialysis; TB = Mycobacterium tuberculosis; WBC = white blood cell.

^a^Pearson’s χ^2^ test; ^b^Fisher's exact test; ^c^Wilcoxon rank-sum (Mann-Whitney) test; ^d^peritonitis episode count excluding peritonitis relapse.

SUPPLEMENTARY FIGURE 1: Kaplan-Meier survival curves for staphylococcal peritonitis


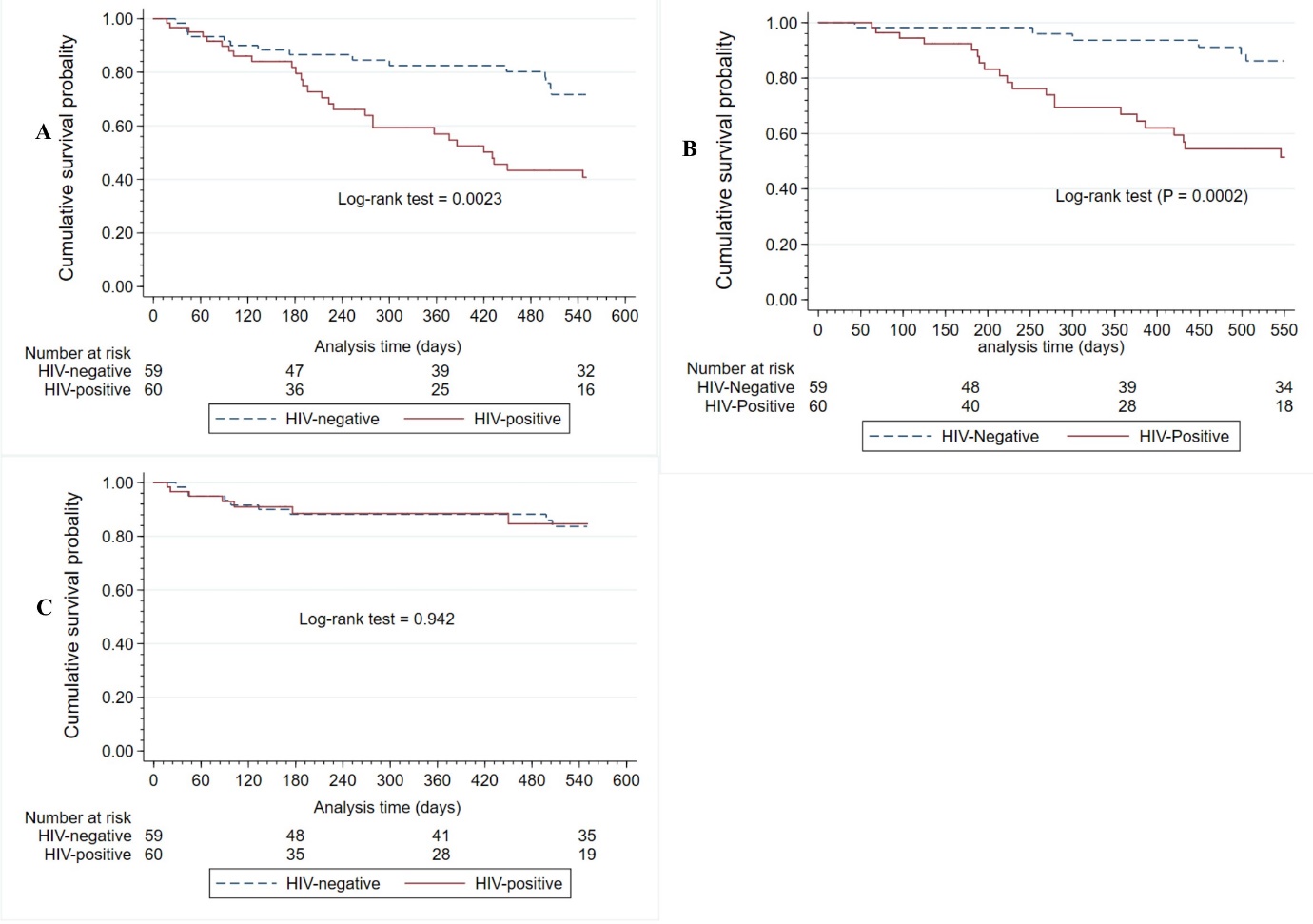


1A — Kaplan-Meier survival curves for staphylococcus species peritonitis free survival according to human immunodeficiency virus infection status censored for death and technique failure.

1B — Kaplan-Meier survival curves for coagulase-negative staphylococci peritonitis free survival according to human immunodeficiency virus infection status censored for death and technique failure.

1C — Kaplan-Meier survival curves for Staphylococcus aureus peritonitis free survival according to human immunodeficiency virus infection status censored for death and technique failure.

HIV = human immunodeficiency virus.
